# Chronic hyperglycemia induced via the heterozygous knockout of Pdx1 worsens neuropathological lesion in an Alzheimer mouse model

**DOI:** 10.1038/srep29396

**Published:** 2016-07-12

**Authors:** Chuang Guo, Shuai Zhang, Jia-Yi Li, Chen Ding, Zhao-Hui Yang, Rui Chai, Xu Wang, Zhan-You Wang

**Affiliations:** 1 College of Life and Health Sciences, Northeastern University, Shenyang, 110819, P. R. China; 2 Basic Medicine Combined with Chinese Traditional Medicine and Western Medicine, Liaoning University of Traditional Chinese Medicine, Shenyang 110847, P. R. China; 3 Department of Endocrinology and Metabolism, Institute of Endocrinology, Liaoning Provincial Key Laboratory of Endocrine Diseases, The First Affiliated Hospital of China Medical University, Shenyang, 110001, P. R. China

## Abstract

Compelling evidence has indicated that dysregulated glucose metabolism links Alzheimer’s disease (AD) and diabetes mellitus (DM) via glucose metabolic products. Nevertheless, because of the lack of appropriate animal models, whether chronic hyperglycemia worsens AD pathologies *in vivo* remains to be confirmed. Here, we crossed diabetic mice (Pdx1^+/−^ mice) with Alzheimer mice (APP/PS1 transgenic mice) to generate Pdx1^+/−^/APP/PS1. We identified robust increases in tau phosphorylation, the loss of the synaptic spine protein, amyloid-β (Aβ) deposition and plaque formation associated with increased microglial and astrocyte activation proliferation, which lead to exacerbated memory and cognition deficits. More importantly, we also observed increased glucose intolerance accompanied by Pdx1 reduction, the formation of advanced glycation end-products (AGEs), and the activation of the receptor for AGEs (RAGE) signaling pathways during AD progression; these changes are thought to contribute to the processing of Aβ precursor proteins and result in increased Aβ generation and decreased Aβ degradation. Protein glycation, increased oxidative stress and inflammation via hyperglycemia are the primary mechanisms involved in the pathophysiology of AD. These results indicate the pathological relationship between these diseases and provide novel insights suggesting that glycemic control may be beneficial for decreasing the incidence of AD in diabetic patients and delaying AD progression.

Increasing evidence suggests that diabetes mellitus (DM) is a non-genetic risk factor for Alzheimer’s disease (AD). Epidemiological studies have suggested that DM increases the risk of AD, and an earlier onset of DM is associated with an increased risk of suffering from AD^1^. Subsequent investigations have demonstrated that individuals with the early stage of DM have a significantly increased risk of developing AD relative to the healthy population^2^. Moreover, postmortem studies that have evaluated the brains of diabetic patients have shown increased amyloid-β (Aβ) deposition and hyperphosphorylated tau compared with that in age-matched controls^3^,^4^, and the brains of patients with AD and diabetes exhibit increased AD pathological changes compared with the brains of non-diabetic AD patients^5^. However, the potential biological mechanisms underlying how DM might accelerate the progression of AD remain unclear.

Extracellular senile plaques (SPs), intracellular neurofibrillary tangles (NFTs), and neuronal loss are neuropathological hallmarks of AD and are used to highlight several primary concerns during AD studies^6^. SPs are largely composed of insoluble Aβ, which is a 4 kDa peptide derived from the proteolytic cleavage of the amyloid-β precursor protein (APP) by type 1 transmembrane protein β-site APP cleavage enzyme 1 (BACE1) and the γ-secretase complex^7^. Tau phosphorylation is essential for the maintenance of microtubular integrity and the dynamics of mature neurons^8^; tau phosphorylation is modulated by several protein kinases, including mitogen-activated protein kinase (MAPK), glycogen synthase kinase-3β (GSK-3β), cyclin-dependent kinase 5 (CDK5), and protein phosphatase 2A (PP2A)^9^,^10^. However, abnormally hyperphosphorylated tau causes the formation of NFTs^11^.

DM is a complex metabolic disorder characterized by chronic hyperglycemia, due to reduced insulin secretion and often in combination with insulin resistance (IR). Recent studies indicated that hyperglycemia has a negative effect on cognitive function and is involved in the pathophysiology common to both diabetes and AD^12^,^13^. Notably, dysregulated glucose metabolism has the potential to increase oxidative stress and the formation of advanced glycation end products (AGEs)^14^, which subsequently increases inflammatory pathway activation^15^. The local inflammation initiated by the activated microglia and reactive astrocytes that surround SPs can lead to neuronal damage^16^. Importantly, in many AD animal models with streptozotocin (STZ) injection-induced diabetes, the formation of both SPs and NFTs increased^10^,^17^. However, consistent with neuropathological studies in patients with AD and DM, a recent study demonstrated that the onset of DM exacerbates memory deficits without an increase in the brain Aβ burden in APP23/ob/ob mice^15^. Therefore, we hypothesize that hyperglycemia and/or glucose tolerance are the leading causes by which DM increases the risk of AD. Working under the assumption that increased insulin rather than glucose is responsible for memory improvement, further studies were performed and demonstrated that insulin administration significantly improved the memory performance in AD^18^,^19^,^20^.

Here, we crossbred animals that have been well characterized and widely used in the studies of both diseases. Mice with a model of AD (specifically, APP/PS1 double transgenic mice) were hybridized with heterozygous knockout of pancreatic duodenal homeobox 1 (Pdx1^+/−^) mice as a model for DM^21^. Our results elucidate the mechanism underlying the pathological relationship between AD and DM.

## Results

### Metabolic features of Pdx1^+/−^/APP/PS1 mice

**Figure 1:**
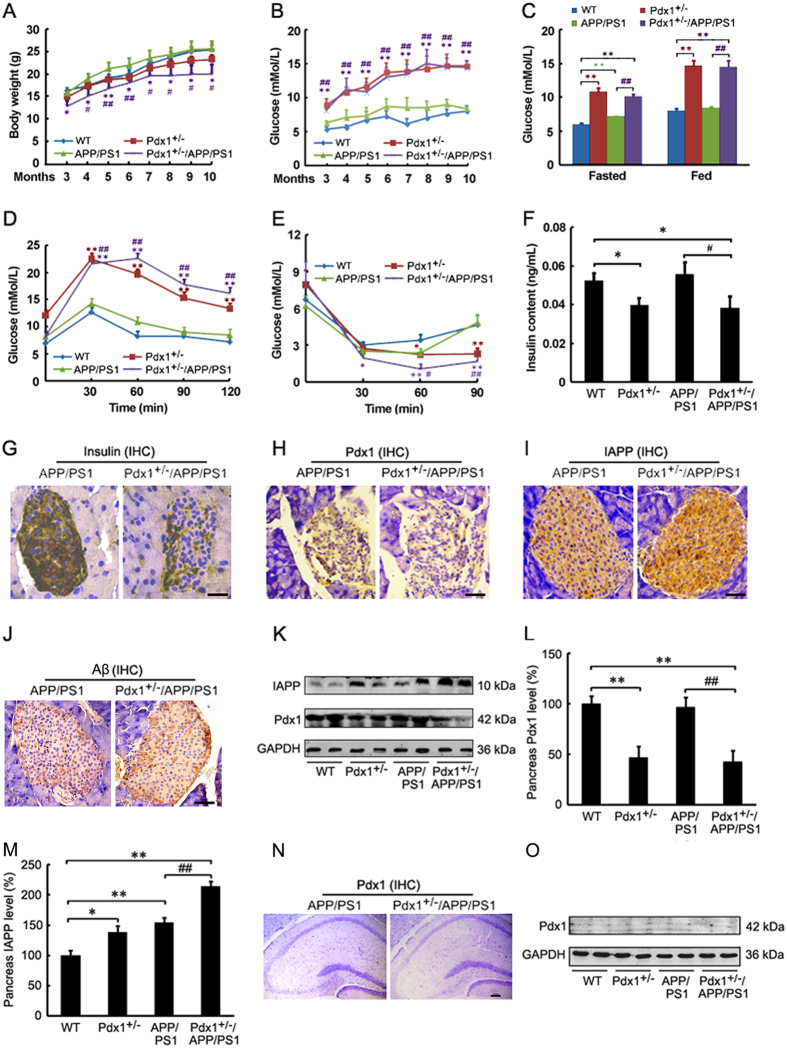
Metabolic features of Pdx1^+/−^/APP/PS1 mice. (**A,B**) Ponderal growth and randomly blood glucose changes in 3- to 10-month-old WT, Pdx1^+/−^, APP/PS1 and Pdx1^+/−^/APP/PS1 mice. (**C**) Blood glucose levels at 41 weeks of age. (**D**) Glucose levels following intraperitoneal injection of 2 g/kg glucose at 12 weeks of age. (**E**) Blood glucose levels during an ITT (0.75 U/kg, 13-week-old mice). (**F**) Serum insulin concentrations at 41 weeks of age. (**G–J**) Pancreatic sections were stained with antisera against insulin/Pdx1/IAPP/Aβ in islets from 10-month-old APP/PS1 and Pdx1^+/−^/APP/PS1 mice for immunohistochemistry. Scale bar = 25 μm. (**K–M**) Western blot analysis showed that the Pdx1 levels were decreased, whereas the IAPP levels were markedly increased in the Pdx1^+/−^/APP/PS1 mouse brain compared with the APP/PS1 mouse brain. GAPDH was used as an internal control. (**N,O**) Immunohistochemistry and Western blot results showed that the Pdx1 protein had not been observed in the hippocampus of mice. Data represent the mean ± S.E. (n = 10). *p < 0.05, **p < 0.01 compared with the WT control group; ^#^p < 0.05, ^##^p < 0.01 compared with the APP/PS1 group.

Pdx1^+/−^ mice exhibit impaired glucose-induced insulin secretion associated with progressive glucose intolerance and the development of diabetes^21^. To determine the potential effect of chronic hyperglycemia on AD-like pathophysiology, we generated an animal model by crossbreeding APP/PS1 and Pdx1^+/−^ mice. The metabolic features and body weight of the double transgenic mice were evaluated by monitoring these variables. The ponderal growth of the Pdx1^+/−^/APP/PS1 mice was lower than that of the original APP/PS1 mice (p** < **0.05 or p** < **0.01; Fig. 1A). Importantly, the Pdx1^+/−^/APP/PS1 mice exhibited worse hyperglycemia with age, and their serum insulin levels were significantly lower than those of the original APP/PS1 mice (p** < **0.05 or p** < **0.01; Fig. 1B,C,F). Moreover, the Pdx1^+/−^/APP/PS1 mice exhibited impaired glucose clearance in GTT compared with that in the APP/PS1 mice (p** < **0.01; Fig. 1D) at 12 weeks of age. In addition, we also demonstrated that the insulin sensitivity measured by ITT was markedly enhanced in the Pdx1^+/−^/APP/PS1 mice compared with the APP/PS1 mice (Fig. 1E) at 13 weeks of age. However, there was no significant difference in the insulin sensitivity or glucose tolerance between the Pdx1^+/−^/APP/PS1 and Pdx1^+/−^ mice (Fig. 1D,E). As expected, the islets in the Pdx1^+/−^/APP/PS1 mice appeared abnormally small with a paucity of insulin-staining β-cells compared with the APP/PS1 mice, as indicated by immunohistochemistry (Fig. 1G). The expression levels of Pdx1 were markedly decreased in the pancreas of the Pdx1^+/−^/APP/PS1 mice compared with the APP/PS1 mice (Fig. 1H,K,L). Interestingly, we observed that IAPP deposition in islets of Pdx1^+/−^/APP/PS1 mice was more pronounced than those of other mice (p** < **0.01; Fig. 1I,K,M), but the positive Aβ-immunoreaction in islets did not differ significantly between the Pdx1^+/−^/APP/PS1 and APP/PS1 mice (Fig. 1J). Moreover, the Pdx1 protein was not observed in the hippocampus as examined by both immunohistochemistry and immunoblot (Fig. 1N,O). These results suggest that Pdx1 deficiency may aggravate the severity of hyperglycemia rather than IR, and that Alzheimer amyloid pathology could also exacerbate diabetes.

### Memory deficits in Pdx1^+/−^/APP/PS1 mice

**Figure 2:**
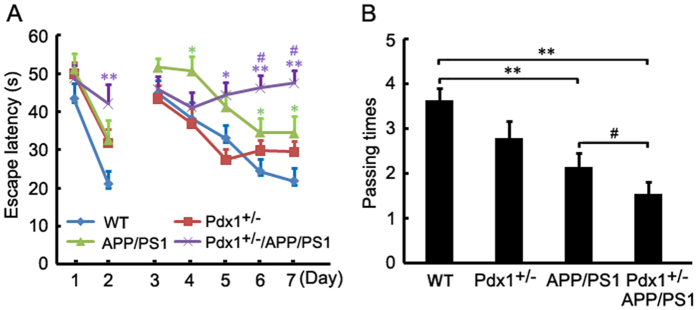
Morris water maze assessment of Pdx1^+/−^/APP/PS1 mice. (**A**) The results of the hidden platform tests did not differ between the Pdx1^+/−^ and WT control groups. The APP/PS1 mice exhibited a significantly longer escape latency than that of the control mice, and the Pdx1^+/−^/APP/PS1 mice exhibited a significantly poorer performance than that of the APP/PS1 mice at 40 weeks of age. (**B**) Memory test in the MWM probe trial without the platform. During the probe trial, the APP/PS1 and Pdx1^+/−^/APP/PS1 mice traveled to the center of the quadrant fewer times than did the WT mice. Note that the deficits in the Pdx1^+/−^/APP/PS1 mice were increased compared with those of the APP/PS1 mice. Data represent the mean ± S.E. (n = 10). *p < 0.05, **p < 0.01 compared with the WT control group; ^#^p < 0.05, ^##^p < 0.01 compared with the APP/PS1 group.

To evaluate whether hyperglycemia affects learning and memory in APP/PS1 mice, the mice were subjected to MWM tests at 40 weeks of age (Fig. 2). The results of the pre-training, visible platform tests for the Pdx1^+/−^ and APP/PS1 mice did not differ from those of the WT mice (Fig. 2A), whereas the Pdx1^+/−^/APP/PS1 mice exhibited a significantly poorer performance, which suggests a possible influence on visual function.

In the hidden platform tests, there were no significant differences between the Pdx1^+/−^ and control mice (Fig. 2A), whereas the APP/PS1 mice exhibited a significantly longer escape latency than that of the control mice (p** < **0.05; Fig. 2A). Notably, compared with the APP/PS1 mice, the Pdx1^+/−^/APP/PS1 mice exhibited a severe learning deficit at this age (p** < **0.05; Fig. 2A).

During the probe trial, the mice in the APP/PS1 and Pdx1^+/−^/APP/PS1 mice explored to the center of the quadrant (where the hidden platform had previously been located) fewer times than the WT mice (p** < **0.01; Fig. 2B). There were no significant differences in the number of travel times between the Pdx1^+/−^ and WT mice (p > 0.05; Fig. 2B). However, the number of travel times of the Pdx1^+/−^/APP/PS1 mice was significantly smaller than that of the APP/PS1 mice (p** < **0.05; Fig. 2B). These results suggest that chronic hyperglycemia exacerbated the cognitive impairment in APP/PS1 mice, whereas hyperglycemia itself did not affect the learning and memory ability or the performance in the test.

### Aβ deposition and synapse loss in Pdx1^+/−^/APP/PS1 mice

**Figure 3:**
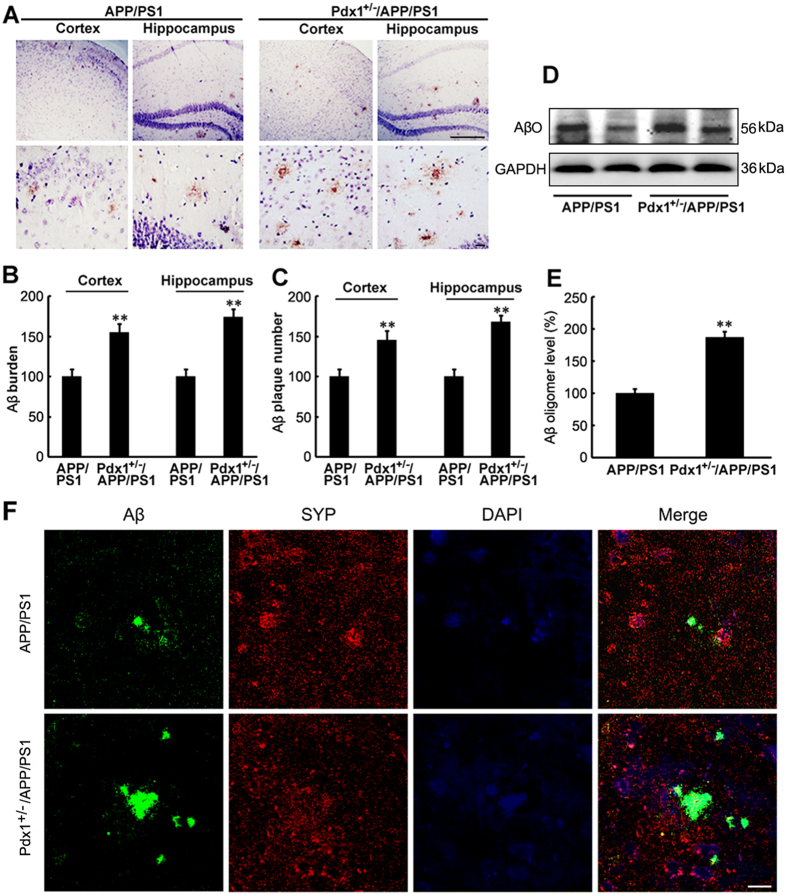
Aβ deposition and synapse loss in Pdx1^+/−^/APP/PS1 mice. (**A**) Immunohistochemical staining indicating the distribution of Aβ plaques in the cortex and hippocampus of the APP/PS1 and Pdx1^+/−^/APP/PS1 mouse brains. (**B,C**) Quantification revealed that hyperglycemia significantly increased the number and area of Aβ plaques in the cortex and hippocampus. Analysis was performed using Image-Pro Plus 6.0 software. (**D,E**) Western blot analysis demonstrated that the Aβ oligomer levels were markedly increased in the Pdx1^+/−^/APP/PS1 mouse brains compared with the APP/PS1 mouse brains. GAPDH was used as an internal control. (**F**) Immunofluorescence labeling and confocal microscopy analysis revealed the distribution and expression of anti-SYP and Aβ in the brain sections of the APP/PS1 mice. Negative stains for SYP indicated synaptic loss. Scale bar = 100 μm. Data represent the mean ± S.E. (n = 10). *p < 0.05, **p < 0.01 compared with the WT control group; ^#^p < 0.05, ^##^p < 0.01 compared with the APP/PS1 group.

To investigate the effects of hyperglycemia on Aβ deposition in APP/PS1 mouse brains, we compared the levels of SP and AβO between the Pdx1^+/−^/APP/PS1 and APP/PS1 mice. Immunohistochemistry indicated that the SP formation was markedly increased in both the cortex and hippocampus of the Pdx1^+/−^/APP/PS1 mice compared with the APP/PS1 one (Fig. 3A–C). Quantitative analyses demonstrated that hyperglycemia significantly increased the number and size of Aβ-immunoreactive SPs in the cortex and hippocampus of the APP/PS1 mouse brains. AβOs in the brains of the Pdx1^+/−^/APP/PS1 mice were examined by immunoblot, as shown in Fig. 3; the levels of AβOs were significantly higher than those in APP/PS1 mice (p** < **0.01; Fig. 3D,E).

We next evaluated whether hyperglycemia affects synapse alterations. As shown in Fig. 3F, the SPs in the Pdx1^+/−^/APP/PS1 mouse brains significantly increased synapse density. We identified a trend of increased SYP loss relative to that in APP/PS1 mice.

These data suggest that the impaired cognitive function in the Pdx1^+/−^/APP/PS1 mice might be related to increases in Aβ deposition, insoluble fractions, and synapse loss in the brain.

### Analysis of the modulation of Aβ signaling mechanisms

**Figure 4:**
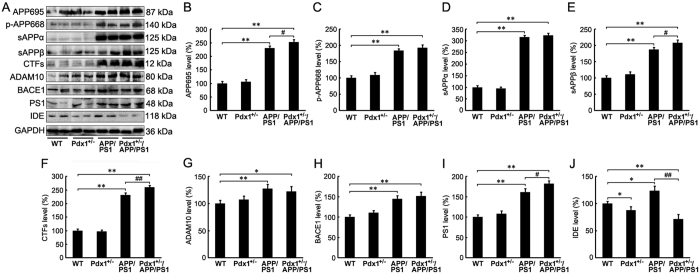
Analysis of the modulation of Aβ signaling mechanisms. (**A**) Western blots showing the protein levels of APP695, p-APP668, ADAM-10, BACE-1, sAPPα, sAPPβ, CTFs, PS1, and IDE in the homogenized brain tissues of Pdx1^+/−^, APP/PS1, Pdx1^+/−^/APP/PS1, and WT littermate mice at 41 weeks of age. GAPDH was used as an internal control. (**B–J**) Quantitative analyses of the immunoreactivities to the antibodies presented in the previous panel. Data represent the mean ± S.E. (n = 10). *p < 0.05, **p < 0.01 compared with the WT control group; ^#^p < 0.05, ^##^p < 0.01 compared with the APP/PS1 group.

Considering the results regarding increased Aβ deposition, we subsequently examined the effects of a hyperglycemic state for 42 weeks in the Pdx1^+/−^/APP/PS1 mice. The APP695 level remained markedly increased in the APP/PS1 group compared with the WT group (p** < **0.01; Fig. 4A,B), and the increase was significantly strengthened by hyperglycemia (p** < **0.05; Fig. 4A,B). The downstream APP processing also resulted in a significant increase in sAPPβ and in the CTF levels compared with those in the APP/PS1 group (p** < **0.05; Fig. 4A,E,F). This alteration in APP processing appeared to be accompanied by an up-regulation of PS1 levels in the brains of the Pdx1^+/−^/APP/PS1 mice (Fig. 4A,I). Notably, IDE, which is involved in Aβ degradation, exhibited robust down-regulation in the brains of the Pdx1^+/−^/APP/PS1 mice compared with the APP/PS1 mice (p** < **0.01; Fig. 4A,J). Furthermore, consistent with previous reported, the IDE levels were found to be greater in APP/PS1 mice than in wild-type littermates, whereas were significantly decreased in the Pdx1^+/−^ group compared with the WT group (Fig. 4A,J). These data suggest that deficiency in insulin might be accompanied by a diminished IDE production that could lead to or aggravate AD.

Therefore, we can infer that hyperglycemia exposure promoted the cerebral processing of APP during amyloidosis and attenuated Aβ clearance, which subsequently resulted in SP formation in the Pdx1^+/−^/APP/PS1 mouse brains.

### Exacerbation of tau pathology in Pdx1^+/−^/APP/PS1 mouse brains

**Figure 5:**
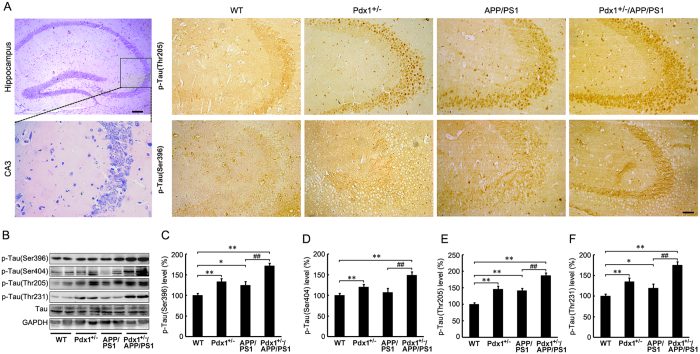
Exacerbation of tau pathology in Pdx1^+/−^/APP/PS1 mouse brains. (**A**) HE staining showing the location of the hippocampal CA3 subfield of the Pdx1^+/−^/APP/PS1 mouse brain. Representative immunohistochemical staining for p-Tau (Thr205)- and p-Tau (Ser396)-positive areas in the hippocampal CA3 subfield of WT, Pdx1^+/−^, APP/PS1, and Pdx1^+/−^/APP/PS1 mice. Scale bar = 60 μm. (**B**) Representative Western blots of total tau and tau phosphorylated at Ser396, Ser404, Thr205, and Thr231 in the homogenized brain tissues of Pdx1^+/−^, APP/PS1, Pdx1^+/−^/APP/PS1, and WT littermate mice at 41 weeks of age. GAPDH was used as an internal control. (**C–F**) Densitometric analyses of the immunoreactivities to the antibodies presented in the previous panel. Data represent the mean ± S.E. (n = 10). *p < 0.05, **p < 0.01 compared with the WT control group; ^#^p < 0.05, ^##^p < 0.01 compared with the APP/PS1 group.

For the next step, we examined the changes in tau pathology following a chronic hyperglycemic state. As shown in Fig. 5A, immunohistochemical staining indicated that phospho-tau (Thr205 and Ser396) immunoproducts were increased in CA3 subfield of hippocampus of the Pdx1^+/−^, APP/PS1, and Pdx1^+/−^/APP/PS1 mice compared with the WT mice. Moreover, the increase of tau phosphorylated at the Thr205 and Ser396 sites were more apparent in the Pdx1^+/−^/APP/PS1 mice than in the APP/PS1 mice. Consistent with the staining, significant increases in the levels of tau phosphorylated at Thr231, Thr205, Ser396, and Ser404 were evident in the hyperglycemia-exposed animals compared with the APP/PS1 mice; there were similar changes in the brains of the Pdx1^+/−^ mice compared with the WT mice (p** < **0.01 or p** < **0.05, respectively; Fig. 5B–F). However, the total tau levels did not differ among the groups, suggesting that hyperglycemia exposure in APP/PS1 mice resulted in increased tau hyperphosphorylation levels (Fig. 5B). These data clearly demonstrate that the chronic hyperglycemic state exacerbates tangle pathologies in the brain.

### Analysis of the modulation of tau hyperphosphorylation signaling mechanisms

**Figure 6:**
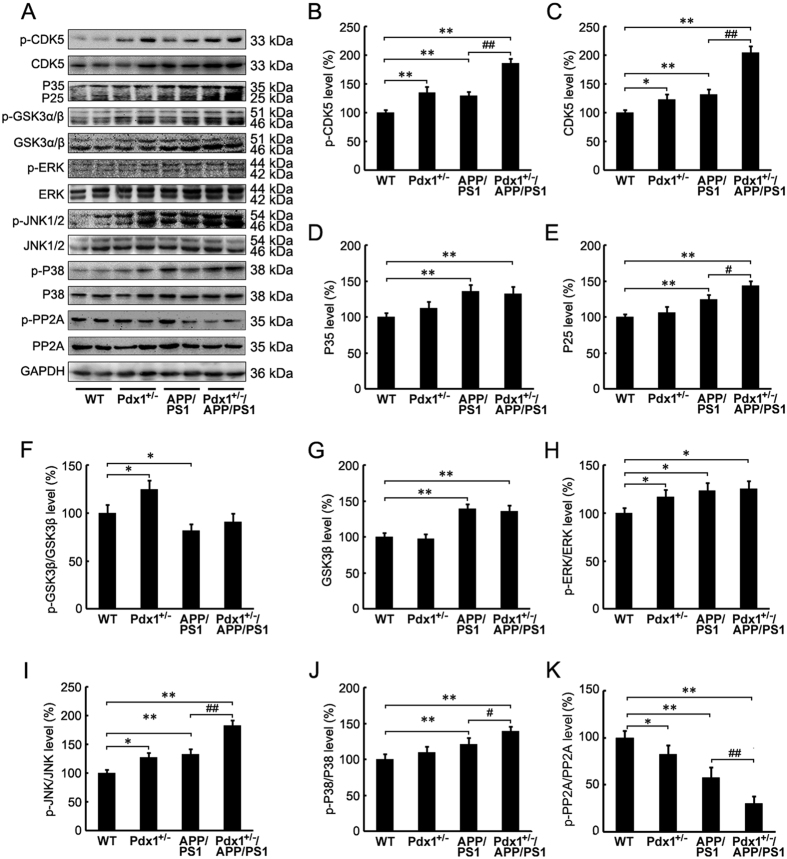
Analysis of the modulation of tau hyperphosphorylation signaling mechanisms. (**A**) Western blots showing the protein levels of GSK3α/β, p-GSK3α/β, ERK1/2, p-ERK1/2, JNK1/2, p-JNK1/2, P38, p-P38, Cdk5, p35, p25 and PP2A in the homogenized brain tissues of Pdx1^+/−^, APP/PS1, Pdx1^+/−^/APP/PS1, and WT littermate mice at 41 weeks of age. GAPDH was used as an internal control. (**B–K**) Quantitative analyses of the immunoreactivities to the antibodies presented in the previous panel. Data represent the mean ± S.E. (n = 10). *p < 0.05, **p < 0.01 compared with the WT control group; ^#^p < 0.05, ^##^p < 0.01 compared with the APP/PS1 group.

To further investigate the molecular mechanism by which the chronic hyperglycemic state induced tau hyperphosphorylation in the brains of the Pdx1^+/−^/APP/PS1 mice, we examined the kinases associated with abnormal tau phosphorylation in the brain. We observed that the brains of the Pdx1^+/−^/APP/PS1 mice exhibited significant increases in p-CDK5 and CDK5, and these increases were accompanied by increased formation of p25 (p** < **0.01 or p** < **0.05, respectively; Fig. 6A–E). In contrast, the GSK3α/β phosphorylation was not significantly different between the APP/PS1 and Pdx1^+/−^/APP/PS1 groups; however, differences in total and p-GSK3α/β were observed between the WT and Pdx1^+/−^ groups as well as between the WT and APP/PS1 groups (Fig. 6A,F,G). No differences in the levels of total ERK, JNK, P38/MAPK or phosphorylated ERK were found among the groups. In contrast, the levels of phosphorylated JNK and P38/MAPK in the brains of the Pdx1^+/−^/APP/PS1 mice were significantly increased compared with those in the APP/PS1 mice (p** < **0.01 or p** < **0.05, respectively; Fig. 6A,H,I,J). In addition, a dramatic inhibition of PP2A activity was induced in the brains of the Pdx1^+/−^, APP/PS1 mice, and the brains of the Pdx1^+/−^/APP/PS1 mice exhibited more severe inhibition relative to that in the APP/PS1 mice (p** < **0.01; Fig. 6A,K). These results suggest that the tau hyperphosphorylation induced by chronic hyperglycemia may be mediated by several active kinases, including CDK5, JNK, and P38 but not GSK3β; furthermore, PP2A inhibition may play an important role.

### Upregulated AGE/RAGE signaling in Pdx1^+/−^/APP/PS1 mouse brains

**Figure 7:**
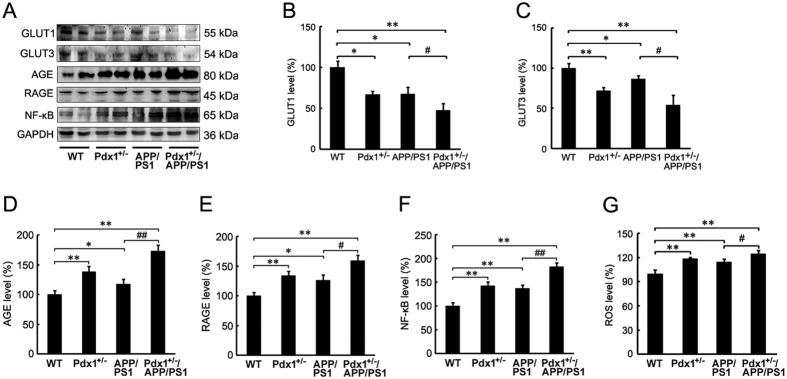
Upregulated AGE/RAGE signaling in Pdx1^+/−^/APP/PS1 mouse brains. (**A**) Western blots demonstrating the protein levels of GLUT1, GLUT3, AGE, RAGE, and NF-κB in the brains of Pdx1^+/−^, APP/PS1, Pdx1^+/−^/APP/PS1, and WT littermate mice at 41 weeks of age. GAPDH was used as an internal control. (**B–F**) Quantitative analyses of the immunoreactivities to the antibodies presented in the previous panel. (**G**) ROS content was increased markedly in the hippocampus of Pdx1^+/−^/APP/PS1 mice compared with APP/PS1 mice. Data represent the mean ± S.E. (n = 10). *p < 0.05, **p < 0.01 compared with the WT control group; ^#^p < 0.05, ^##^p < 0.01 compared with the APP/PS1 group.

Impaired cerebral glucose metabolism is a pathophysiological feature in AD and its attack predates pathological changes even for decades^22^. Accordingly, we investigated whether the increased hyperphosphorylation of tau involved in the reduced glucose transporter (GLUT)1 and GLUT3, which were considered to play essential roles in the modulation of brain glucose transportation^22^. We found that the GLUT1 and GLUT3 levels were statistically decreased in the brains of the Pdx1^+/−^ and APP/PS1 mice compared with those in the WT mice, and the decrease of GLUT1 and GLUT3 levels were more severe in the Pdx1^+/−^/APP/PS1 mice than in the APP/PS1 mice (Fig. 7A–C; p** < **0.05 or p** < **0.01, respectively). In fact, apart from decreased GLUT 1 and GLUT 3, elevated AGEs also could occur and even play significant roles in AD^22^. AGE/RAGE activation has been reported to precede the steep increase in cerebral Aβ and the formation of plaques^23^, to accelerate Aβ deposition^24^, and to induce the production of reactive oxygen species (ROS) and the subsequent activation of NF-κB^14^,^25^. As presented in Fig. 7, the levels of AGE, RAGE, and NF-κB were significantly increased in the brains of the Pdx1^+/−^, APP/PS1, and Pdx1^+/−^/APP/PS1 mice relative to the WT mice, as assessed by Western blotting. The brains of the Pdx1^+/−^/APP/PS1 mice contained much higher levels of these proteins relative to those in the APP/PS1 mice (Fig. 7A,D,E,G; p** < **0.05 or p** < **0.01, respectively). In addition, the change pattern of ROS content was the same as that of NF-κB (Fig. 7G).

### Increased neuroinflammation in Pdx1^+/−^/APP/PS1 mouse brains

**Figure 8:**
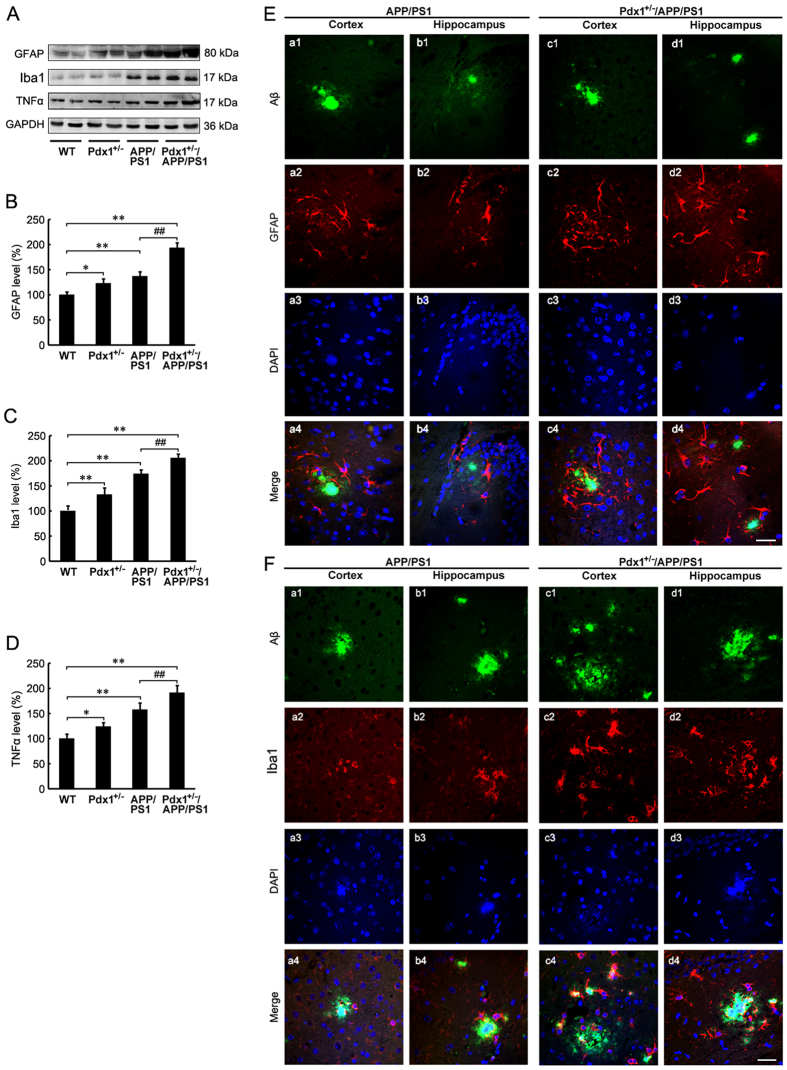
Increased neuroinflammation in Pdx1^+/−^/APP/PS1 mouse brains. (**A**) Western blots demonstrating the protein levels of GFAP, Iba1 and TNFα in the brains of Pdx1^+/−^, APP/PS1, Pdx1^+/−^/APP/PS1, and WT littermate mice at 41 weeks of age. GAPDH was used as an internal control. (**B–D**) Quantification revealed that the levels of GFAP, Iba1 and TNFα were significantly increased in the brains of the Pdx1^+/−^/APP/PS1 mice compared with the APP/PS1 mice. Immunofluorescence labeling and confocal microscopy analysis showing the distribution and expression of Aβ (a1-d1) and GFAP (**E**) and Iba1 (F) (a2-d2) in the cortex and hippocampus of the APP/PS1 and Pdx1^+/−^/APP/PS1 mouse brains. The images are representative of three independent experiments. Scale bar, 20 μm. Data represent the mean ± S.E. (n = 10). *p < 0.05, **p < 0.01 compared with the WT control group; ^#^p < 0.05, ^##^p < 0.01 compared with the APP/PS1 group.

Inflammatory reactions are a consistent characteristic of AD, and the activation of RAGE induces oxidative stress and inflammation^26^. In this study, we demonstrated that relative to the APP/PS1 mice, the Pdx1^+/−^/APP/PS1 mice exhibited significantly increased GFAP and Iba1 immunoreactivities using double labeling with Aβ in the brain sections. Further, increased gliacytes showed positive staining around the plaques (Fig. 8E,F). These findings indicate activation of astrocytes and microglia, respectively. To further assess reactive astrogliosis, we examined the expression levels of GFAP and Iba1 in the mouse brains. We observed that the Pdx1^+/−^/APP/PS1 mice exhibited more increased GFAP, Iba1 and TNFα contents (p** < **0.01; Fig. 8A–D), whereas the mRNA levels of IL-1β and IL-6 were not significantly increased in the brains of the Pdx1^+/−^/APP/PS1 mice relative to those in the APP/PS1 mice (data not shown).

## Discussion

Our previous studies have demonstrated that diabetes could accelerate the development of the cerebral amyloidosis connected to AD pathology in a mouse model of combined insulin-deficient diabetes and AD via STZ injection^10^. Here, we developed an animal model that exhibited both diabetes and AD by crossing APP/PS1 and Pdx1^+/−^ mice. Our model exhibited a marked increase in blood glucose levels without IR. The current study demonstrates that chronic hyperglycemia not only increased the SP formation but also triggered tau hyperphosphorylation and synapse loss in the brain, thus potentiating the cognitive dysfunction in the Pdx1^+/−^/APP/PS1 mice.

Pdx1 is a transcriptional factor essential for the development of the pancreas and foregut^27^. Importantly, heterozygous mutations of the *Pdx1* gene in humans are associated with maturity-onset diabetes of the young type 4^28^. Previous studies have shown that a systemic heterozygous Pdx1 knockout mouse is characterized by glucose intolerance and causes diabetes with increasing age^21^,^29^. Although the Pdx1 gene is expressed in both the developing brain and the adult hypothalamus of Pdx1-Cre mice, no information about its production was available in developed brains^30^,^31^. Pdx1^+/−^ mice were therefore used to characterize and define a highly relevant animal model for studying the pathophysiology of the type of diabetes that is primarily caused by pancreatic defects. However, absolute insulin levels do matter and reduced insulin levels can also be predicted to impair long-term potentiation and cognitive function, in particular in the immature brain^32^. As a predominant clinical feature of diabetes, hyperglycemia is inversely correlated with mild cognitive impairment in AD^33^,^34^,^35^,^36^. Furthermore, AD is associated with hyperglycemia^37^,^38^, which indicates that hyperglycemia may play a role in cognitive decline and AD pathogenesis. To address this important issue, Pdx1^+/−^ mice were used in our study. The GTT and ITT results did not significantly differ between the Pdx1^+/−^ and Pdx1^+/−^/APP/PS1 mice, whereas the Pdx1^+/−^/APP/PS1 mice exhibited significantly increased fasting blood glucose levels, markedly decreased serum insulin levels, and markedly increased responses to glucose or insulin challenges compared with those of their APP/PS1 littermates. The marked effects of the loss of a single Pdx1 allele on the progressive development of glucose intolerance and impaired glucose-stimulated insulin secretion indicate that Pdx1^+/−^ mice may be a reasonable hyperglycemia model for analyzing AD pathogenesis.

In this study, we demonstrate that Pdx1^+/−^ mice exhibit worsening of cognitive deficits of the APP/PS1 mouse; however, the Pdx1^+/−^ mice did not exhibit a significant deterioration in memory performance compared with that of the WT mice. Because DM has been widely implicated in cognition and AD, we cannot exclude the possibility that our observations in the MWM tests could, at least in part, be attributed to the chronic hyperglycemia in this animal model; however, a similar outcome was observed in STZ-induced diabetes, as previously described^39^.

It remains unknown whether hyperglycemia triggers altered APP processing and the subsequent development of clinical AD pathologies. In humans, a recent study using neuroimaging techniques demonstrated that IR is not associated with amyloid deposits^40^, which was similar to the results of previous autopsy studies^37^. Recent data obtained from cross-mated APP23-ob/ob mice indicated the absence of an increase in brain Aβ levels^15^. In this study, we found significantly increased Aβ accumulation in the brains of Pdx1^+/−^APP/PS1 mice. We also observed a significant difference between the APP/PS1 and Pdx1^+/−^/APP/PS1 mice in the PS1 levels, the total amount of CTF, and the APP proteolytic processing that is involved in Aβ production; these findings confirm the direct involvement of this proteolytic pathway in the observed biological effects of hyperglycemia that were also found in this mouse model. Furthermore, the Pdx1^+/−^/APP/PS1 mouse brain had reduced levels of IDE, which is involved in the degradation of the Aβ peptide^41^. This reduction could represent an additional mechanism for the increased SP. Taken together, our results indicate that chronic hyperglycemia participates in enhanced Aβ deposition through increased Aβ production and suppressed Aβ clearance in Pdx1^+/−^/APP/PS1 mice.

As previously reported, in addition to Aβ pathology, abundant intracellular NFTs are also present^8^. In this study, we compared the tau phosphorylation levels at several known major phosphorylation sites (Ser396, Ser404, Thr205, and Thr231) in the brains of the DM and control mice. We observed that the mean tau phosphorylation levels at these sites were increased in the DM mice compared with the control cases. Interestingly, we determined that although tau is hyperphosphorylated in both groups, the complication of AD with hyperglycemia exacerbated the tau phosphorylation levels compared with those for AD alone. Regarding this close relationship between DM and AD, increased tau phosphorylation has been consistently demonstrated in studies that used various animal models^42^,^43^,^44^. Therefore, chronic hyperglycemia might not only increase the risk for AD via the promotion of tau phosphorylation but also accelerate AD via the exacerbation of tau hyperphosphorylation at critical, abnormal phosphorylation sites.

GSK3 is a key molecule downstream of the insulin signaling pathway. Several studies have demonstrated that the activation of GSK3α/β is closely linked to the mechanisms by which STZ-induced dysfunction of insulin cascades promotes the formation of SPs and NFTs^17^,^45^. An unexpected observation in the present study was that chronic hyperglycemia increased the GSK3 phosphorylation levels in the Pdx1^+/−^ mouse brains compared with the WT mouse brains. This change may inhibit GSK3 activity, but there is not a significant difference in the ratio of p-GSK3β/GSK3β between the pure APP/PS1 and the Pdx1^+/−^/APP/PS1 groups. Interestingly, an increase in p-GSK3β was observed in insulin knockout mice^46^, and the inhibition of GSK-3 facilitates the induction of long-term potentiation in mice overexpressing GSK-3^47^. In fact, multiple insulin receptor signaling pathways other than GSK3, such as impairments in AβO clearance, could be involved in the cognitive impairment^48^. Here, we demonstrated that hyperglycemia specifically affected Cdk5 kinase, whose activation is regulated by its binding to the activator proteins p35 and p25. The phosphorylation and steady-state protein levels of Cdk5, as well as the p25 levels, were significantly increased in the Pdx1^+/−^ mice, which suggests that the activation of this kinase is responsible for the changes in tau phosphorylation. ERKs, JNKs and P38 MAPK comprise a group of MAPK serine-threonine kinases^49^,^50^; the activation of these kinases has been demonstrated to contribute to tau hyperphosphorylation, which, in turn, participates in AD pathophysiological alterations^51^. Here, our findings indicated that ERK1/2, JNK and P38 MAPK signaling were activated in the Pdx1^+/−^ mouse brains, and this activation may represent an important molecular mechanism responsible for chronic hyperglycemia. An *in vitro* high glucose binding assay suggested that MAPKs are involved in AD pathology^52^. We also examined the change in PP2A, which is the most important phosphatase involved in tau dephosphorylation and is specifically decreased in AD brains^53^. We observed that hyperglycemia decreased the level of PP2A, which suggests that chronic hyperglycemia may inhibit the activity of PP2A in Pdx1^+/−^ mouse brains and is consistent with the finding that PP2A activity is reduced in AD brains^53^,^54^. Thus, we speculate that chronic hyperglycemia may augment tau hyperphosphorylation through the activation of CDK5, JNK and P38 MAPK signaling and the inhibition of PP2A activity, rather than through GSK3.

GLUT1 and GLUT 3 are considered to play fundamental roles in the regulation of brain glucose transportation and in the pathogenesis of AD^22^,^55^,^56^. Here, we confirmed that GLUT1 and GLUT3 levels were significantly decreased, especially in Pdx1^+/−^/APP/PS1 mouse brains, suggesting that impaired cerebral glucose metabolism by Pdx1 deficiency might contribute to the pathological dysfunction of the brain in AD^22^. Many studies suggest that hyperglycemia induces the creation of AGEs through a non-enzymatic reaction of glucose and other carbohydrates with stable protein complexes, whose abnormal formation and accumulation occur during normal brain aging but are accelerated by diabetes^57^. Studies have reported that diminished GLUTs and AGEs accumulate in SPs and NFTs, and AGEs may also accelerate Aβ deposition^22^,^24^. Therefore, accumulated AGEs may be an important factor shared by DM and AD. AGEs are metabolized through the activation of RAGE. The interaction of AGEs with RAGE promotes the formation of ROS^25^ and mediates the amplification of inflammatory responses^58^,^59^. ROS are cytotoxic byproducts of normal mitochondrial metabolism. Nevertheless, excessive ROS levels may cause oxidative stress and mitochondrial dysfunction, likely as a link between brain inflammation and defective insulin signaling^60^. In fact, it has been recently proposed that AβOs play a key mechanism leading to excessive ROS production and Ca2^+^-related mitochondrial dysfunction, a condition that has been implicated in both T2D and AD^61^,^62^,^63^. Notably, RAGE also binds to Aβ peptides, which causes an increase in the transport of Aβ from the blood to the brain^23^,^58^,^64^, and RAGE is overexpressed in the brain of AD patients^65^. Moreover, RAGE can induce its own expression through the activation of the transcription factor NF-κB^25^. In this study, we demonstrated that the AβOs, AGE and RAGE levels are markedly enhanced in Pdx1^+/−^/APP/PS1 mouse brains. Thus, increased NF-κB protein and ROS were also observed in Pdx1^+/−^ and Pdx1^+/−^/APP/PS1 mice. Previous studies have demonstrated a significant correlation between SP formation and the activation of microglia and astrocytes in AD brains^16^,^66^. As expected, significantly greater Iba-1 and GFAP immunoreactivity was observed in the Aβ deposits of the Pdx1^+/−^/APP/PS1 mice compared with that in the APP/PS1 mice. These results suggest that the activation of the AGE/RAGE axis and the inflammatory changes induced by chronic hyperglycemia may contribute to the increased AD pathology in this model.

In summary, the present study demonstrated that Pdx1^+/−^/APP/PS1 mice exhibit enhanced cognitive decline, Aβ plaque deposition, tau hyperphosphorylation, the loss of synaptic spine protein, and activation of microglia and astrocytes. Our data obtained from cross-mated Pdx1^+/−^/APP/PS1 animals clearly demonstrated the effect of chronic hyperglycemia on AD pathology. The aggravated AD pathology in the DM model suggests that an important pathogenic factor closely related to hyperglycemia plays a critical role in AD pathology^67^, and investigation of this factor will provide insight for designing a strategy to prevent and treat AD.

## Materials and Methods

### Animals

The APP/PS1 mice were originally obtained from Jackson Laboratory. The Pdx1^+/−^ mice, a model of DM, were generated by gene targeting in embryonic stem cells by Cyagen Biosciences, China, as previously described^27^. The animals were maintained under standard conditions. We subsequently intercrossed these mice to generate Pdx1^+/−^, APP/PS1, Pdx1^+/−^/APP/PS1, and WT littermate mice. All mice had the same genetic background (C57BL/6). The animals’ general health and body weights were monitored monthly. The animals were aged up to 40 weeks, and 10 animals/group were included in the studies. All the experimental procedures were approved by the Laboratory Animal Ethical Committee of Northeastern University and performed in strict accordance with the People’s Republic of China Legislation Regarding the Use and Care of Laboratory Animals.

### Metabolic measurements

Blood glucose measurements were performed during fasted (for 12 hours) and randomly fed states at approximately the same time. For glucose tolerance tests (GTTs), twelve-week-old animals were fasted for 12 hours and were administered an intraperitoneal (i.p.) injection of glucose (2 g/kg, Sigma, dissolved in sodium citrate buffer). For insulin tolerance tests (ITTs), thirteen-week-old mice were fasted for 6 hours prior to an i.p. injection of 0.75 U/kg insulin. Blood samples were subsequently obtained by tail prick, and the blood glucose levels were measured using a handheld blood glucose meter at various time points.

### Morris water maze (MWM)

Forty-week-old mice were trained and tested in a MWM as previously described^10^. Finally, the recorded data were analyzed using a computer program (ZH0065; Zhenghua Bioequipment).

### Tissue preparation

After the MWM tests, the mice were anesthetized with sodium pentobarbital (50 mg/kg, i.p.), and venous blood was collected from the retro-orbital sinus. The animals were subsequently sacrificed via decapitation. The brains were quickly removed and dissected in half. One half was fixed in 4% paraformaldehyde in PBS at 4 °C overnight. The fixed tissues were routinely processed for paraffin embedding, and sections (5 μm) were prepared for immunohistochemical or haematoxylin-eosin staining. The other half was frozen at −80 °C for biochemical analyses.

### Sandwich ELISA

The serum insulin levels were measured using mouse insulin ELISA kits (Chemicon), according to the manufacturer’s instructions. The absorbance was measured using a BIO-RAD 3550-UV microplate reader.

### Immunohistochemistry and immunofluorescence

Antigen retrieval from paraffin sections was achieved by boiling in citric acid buffer for 3 minutes in a microwave oven. The sections were incubated with primary antibodies, rabbit anti-insulin or anti-Pdx1 (1:400; CST), rabbit anti-Amylin (IAPP, 1:200; Ruiying Biological), mouse anti-Aβ (1:500; Sigma), rabbit anti-tau-p-Ser396 or anti-tau-p-Thr205 (1:600; Abcam), rabbit anti-GFAP (1:100; Santa Cruz), rabbit anti-Iba1 (1:100; Abcam), rabbit anti-synaptophysin (SYP, 1:200; Abcam), subsequently incubated with donkey anti-mouse IgG conjugated to fluorescein isothiocyanate and Texas-Red donkey anti-rabbit IgG secondary antibodies (1:200; Jackson), and then incubated with either DAPI for double immunofluorescence or with anti-mouse/rabbit IgG (1:200) conjugated to HRP and then with 0.025% DAB for detection as previously described^39^. The images were observed using a confocal laser scanning microscope (SP8, Leica).

### Western blotting

Homogenized cortex, hippocampus and pancreas tissues of mouse at 41 weeks of age were lysed in RIPA buffer supplemented with a protease inhibitor cocktail (Sigma) and were processed for immunoblot analysis as previously described^39^. The total protein lysate (50 μg) was fractionated via 8–12% SDS PAGE and transferred to polyvinylidene fluoride membranes. Primary antibodies (see Table 1) were used. Aβ oligomer (AβO) was checked under nondenaturing conditions. Immunoblots were washed and treated with the appropriate species of HRP-conjugated secondary antibody (1:5000), and immunoreactive bands were visualized by enhanced chemiluminescence using the ChemiDoc XRS+ system and the accompanying Quantity One software.

### Assay for ROS formation

ROS levels in the hippocampus tissues homogenates were analyzed using 2′,7′-dichlorofluorescein diacetate (DCFH-DA) according to the manufacturer’s instructions (Jiancheng Biology, Nanjing, China). DCF fluorescence was monitored at 525 nm emission using a microplate reader (Synergy/H1, BioTek).

### Statistical analyses

The results were expressed as the mean ± standard error of the mean (SEM). Repeated measures analysis of variance (ANOVA) was performed for the MWM tests; differences among the means were evaluated with multivariable ANOVA. Other comparisons were analyzed by two-way ANOVA followed by post hoc Bonferroni tests when appropriate. All data were analyzed using SPSS 16.0 software, and differences were assumed to be statistically significant if p** < **0.05.

## Additional Information

**How to cite this article**: Guo, C. *et al.* Chronic hyperglycemia induced via the heterozygous knockout of Pdx1 worsens neuropathological lesion in an Alzheimer mouse model. *Sci. Rep.* **6**, 29396; doi: 10.1038/srep29396 (2016).

## Figures and Tables

**Table 1:** Primary antibodies used.

Antibody	Dilution	Source
rabbit anti-amyloid oligomer	1:500	Millipore
rabbit anti-APP695	1:4000	Chemicon
rabbit anti-p-APP Thr668	1:1000	CST
rabbit anti-APP-C-terminal fragments (CTFs)	1:4000	Chemicon
rabbit anti-ADAM10	1:1000	Millipore
rabbit anti-AGEs	1:500	Bioss
rabbit anti-BACE1	1:1000	Sigma
rabbit anti-CDK5	1:1000	Abcam
rabbit anti-p-CDK5 (Tyr15)	1:1000	Abcam
rabbit anti-p-ERK	1:1000	CST
rabbit anti-ERK	1:1000	CST
rabbit anti-glial fibrillary acidic protein (GFAP)	1:500	Santa Cruz
rabbit anti-GLUT1	1:800	BBI Life Sciences
rabbit anti-GLUT3	1:500	BBI Life Sciences
rabbit anti-GSK3α/β	1:1000	CST
rabbit anti-p-GSK3α (Ser21)/3β (Ser9)	1:1000	CST
rabbit anti-IAPP	1:1000	Ruiying Biological
rabbit anti-ionized calcium-binding adaptor molecule 1 (Iba1)	1:1000	Wako
goat anti-Insulin Degrading Enzyme (IDE)	1:400	Santa Cruz
rabbit ani-p-JNK	1:1000	CST
rabbit anti-JNK	1:1000	CST
rabbit anti-NF-κB p65	1:1000	Santa Cruz
rabbit anti-p35/25	1:800	CST
rabbit anti-p-P38	1:500	Santa Cruz
rabbit anti-P38	1:500	Santa Cruz
rabbit anti-p-PP2A (Tyr307)	1:1000	Abcam
rabbit anti-PP2A	1:1000	Abcam
rabbit anti-presenilin 1 (PS1)	1:800	Millipore
rabbit anti-RAGE	1:1000	Abcam
mouse anti-sAPPα	1:500	IBL
mouse anti-sAPPβ	1:500	IBL
mouse anti-tau-p-Thr231	1:1000	Invitrogen
rabbit anti-tau-p-Thr205	1:1000	Abcam
rabbit anti-tau-p-Ser396	1:1000	Abcam
rabbit anti-tau-p-Ser404	1:1000	CST
rabbit anti-tau	1:400	Abcam
rabbit anti-TNFα	1:1000	Abcam
mouse anti-GAPDH	1:5000	Kangchen Biotech
